# Production of COx-Free Hydrogen and Few-Layer Graphene Nanoplatelets by Catalytic Decomposition of Methane over Ni-Lignin-Derived Nanoparticles

**DOI:** 10.3390/molecules27020503

**Published:** 2022-01-14

**Authors:** Qiangu Yan, Timothy Ketelboeter, Zhiyong Cai

**Affiliations:** 1 Ligwood LLC, Madison, WI 53719-2380, USA; yanqiangu@gmail.com; 2 Forest Products Laboratory, One Gifford Pinchot Drive, Madison, WI 53726-2398, USA; timothy.ketelboeter@usda.gov

**Keywords:** COx-free hydrogen, few-layer graphene nanoplatelets, catalytic decomposition of methane, graphene-encapsulated nickel nanoparticles, Ni-lignin nanocomposite

## Abstract

Nickel (Ni)-lignin nanocomposites were synthesized from nickel nitrate and kraft lignin then catalytically graphitized to few-layer graphene-encapsulated nickel nanoparticles (Ni@G). Ni@G nanoparticles were used for catalytic decomposition of methane (CDM) to produce COx-free hydrogen and graphene nanoplatelets. Ni@G showed high catalytic activity for methane decomposition at temperatures of 800 to 900 °C and exhibited long-term stability of 600 min time-on-stream (TOS) without apparent deactivation. The catalytic stability may be attributed to the nickel dispersion in the Ni@G sample. During the CDM reaction process, graphene shells over Ni@G nanoparticles were cracked and peeled off the nickel cores at high temperature. Both the exposed nickel nanoparticles and the cracked graphene shells may participate the CDM reaction, making Ni@G samples highly active for CDM reaction. The vacancy defects and edges in the cracked graphene shells serve as the active sites for methane decomposition. The edges are continuously regenerated by methane molecules through CDM reaction.

## 1. Introduction

Hydrogen proton-exchange membrane fuel cells (H_2_-PEMFCs) are promising energy conversion devices for electric vehicles and portable appliances. However, the platinum catalysts in the electrodes of PEMFCs are easily poisoned by trace amounts of carbon monoxide (CO) in the hydrogen. Therefore, only high purity (100 ppm or less of CO) hydrogen is desired for use in H_2_-PEMFC devices [1]. More than 50% of hydrogen is presently produced from steam reforming of natural gas, which simultaneously generates CO and CO_2_ (Equation (1)).

CH_4_ + H_2_O → CO + 3 H_2_, Δ*H*_298_ = 206 kJ/mol (1)

COx (CO and CO_2_) must be removed through a series of purification processes before obtaining high purity hydrogen [2]. First, the water-gas shift reaction (WGSR) is performed to convert CO to CO_2_ over a catalyst (usually an iron oxide catalyst) (Equation (2)):

CO + H_2_O → CO_2_ + H_2_, Δ*H*_298_ = −41 kJ/mol (2)

Then, CO_2_ is separated from hydrogen using the pressure swing adsorption (PSA) process, where adsorbent materials (zeolites, activated carbon, molecular sieves, etc.) are used to remove CO_2_ at high pressure [3]. Significant amounts of energy and materials are consumed in these purification processes.

In the past decade, catalytic decomposition of methane (CDM) (Equation (3)) has been extensively explored to replace the steam reforming method for hydrogen production [2]. Since the CDM reaction does not produce CO or CO_2_, the WGSR and PSA steps are eliminated from the hydrogen production process.

CH_4_ → C + 2H_2_, Δ*H*_298_ = 75.6 kJ/mol (3)

Catalysts used in CDM are categorized as metal- and carbon-based materials [4]. Metal-based catalysts can be classified into supported catalysts and non-supported catalysts. Transition metal catalysts, especially nickel, cobalt, and iron-based catalysts, have received extensive attention for use in the CDM process due to their low cost, good catalytic activity, and stability [5]. Among them, Ni is an excellent catalyst due to its sulfur resistance compared to the other metals; however, Ni catalysts quickly deactivate due to rapid aggregation and carbon encapsulation at temperatures above 600 °C [4].

Various carbon materials have been used for catalytic decomposition of methane including a wide range of activated carbons (AC), carbon blacks (CB), graphite, glassy carbon, acetylene black, graphite, diamond powder, carbon nanotubes (CNT), and fullerenes [6]. The most widely studied carbon materials for use in CDM are activated carbons and carbon blacks because of their activity and good stability [6,7]. Operating parameters that have been studied to determine the activity and long-term stability of these catalysts are temperature range, pressure, and methane flow rate [8,9]. 

Lignin is a major component of lignocellulosic biomass and the most abundant aromatic biopolymer. It contains up to 65 wt% carbon and has been used as an important carbon resource to produce carbon fibers [10], carbon foams [11], and graphene-based nanomaterials [12]. Graphene oxide has been reported as a component of the catalysts used in photocatalytic hydrogen production [13]. Graphene-based core-shell nanostructures have extensively been studied for use as catalysts in hydrogen production [14] and use in fuel cells [15]; however, there is presently no study on graphene-based core-shell nanostructures for production of hydrogen through the CDM reaction.

In this study, Ni-lignin composites were prepared using the coprecipitation method then catalytically graphitized to few-layer graphene-encapsulated nickel nanoparticles (Ni@G). These Ni@G nanoparticles were used for catalytic decomposition of methane (CDM) to produce COx-free hydrogen and graphene nanoplatelets. During the CDM reaction process, Ni@G nanoparticles were cracked, few-layer graphene shells were peeled off the nickel cores, and the encapsulated nickel nanoparticles were simultaneously exposed to the reaction atmosphere. Both the uncovered nickel nanoparticles and the cracked graphene shells may participate the CDM reaction, making Ni@G samples highly active for CDM reaction. This work focuses on studying the effects of the process conditions on hydrogen production and the yield and structure of graphene materials.

## 2. Materials and Methods

### 2.1. Materials

Powdered kraft lignin (BioChoice, Plymouth, NC, USA) was supplied by Domtar. The ash content in the kraft lignin was 1.65 wt% determined using ASTM D1102 standard. The number average molecular weight (M_n_) and weight average molecular weight (M_w_) of kraft lignin were determined to be M_n_ = 988 ± 56 g/mol and M_w_ = 6672 ± 315 g/mol, respectively, measured using gel permeation chromatography (GPC). Lignin elemental analysis was performed with an elemental analyzer. Nickel nitrate hexahydrate (Ni(NO_3_)_2_⋅6H_2_O) and tetrahydrofuran (THF, anhydrous, ≥99.9%, inhibitor-free) were purchased from Sigma-Aldrich, Inc. (St. Louis, MO, USA).

### 2.2. Preparation of Ni-Lignin Composites

Ni-lignin composites were prepared using the co-precipitation method. Five solutions of nickel nitrate were prepared by adding 13.0 g, 26.6 g, 56.2 g, 89.2 g, and 126.4 g nickel nitrate hexahydrate to 15 mL, 30 mL, 60 mL, 90 mL, and 120 mL of DI water, respectively, in individual 500 mL glass beakers and stirring for 30 min. Each of these five nickel nitrate solutions was added dropwise to its respective tetrahydrofuran and kraft lignin solution (100 g lignin in 100 mL tetrahydrofuran) and the final mixtures were all stirred for 2 h. The mixtures were naturally dried at room temperature for one week. The prepared Ni-lignin composites were labelled as 2.5% Ni-lignin, 5% Ni-lignin, 10% Ni-lignin, 15% Ni-lignin, and 20% Ni-lignin, respectively.

### 2.3. Pretreatment of Ni-Lignin Composites

Thermal decomposition of Ni-lignin composites has previously been observed as a strongly exothermic process. A significant amount of heat is released during the reaction which may trigger a thermal runaway situation [16]. Therefore, the dried Ni-lignin composites were first thermally decomposed in a muffle furnace before loading into a reactor for the catalytic graphitization process. Nitrogen gas was first introduced into the furnace at a flow rate of 100 mL/min for 30 min. The furnace temperature was increased to 300 °C at a rate of 2.5 °C/min and held at 300 °C for 0.5 h, then naturally cooled to ambient temperature under nitrogen flow. The decomposed sample was loaded into a ball mill machine and ground at 1000 rpm for 10 min.

### 2.4. Catalytic Carbonization of Ni-Lignin Composites to Graphene-Encapsulated Nickel Nanoparticles (GENNs, Labelled as Ni@G)

Fifty grams (50 g) of the decomposed Ni-lignin samples was each packed in the middle of a 1 inch OD ceramic tubular reactor. The carrier gas–argon (99.99% purity) was first introduced into the reactor at a flow rate of 100 mL/min for 30 min. The reactor was heated at a temperature-programmed rate of 10 °C/min to a carbonization temperature (600, 700, 800, or 900 °C) and held at the carbonization temperature for 1 h. The furnace was cooled down by 10 °C/min to room temperature under an argon flow. An on-line Hiden QGA quantitative gas analysis system (Hiden Analytical, Livonia, MI, USA) was used to measure gaseous products during the graphitization process. The signals from the mass spectra of 2, 15, 28, and 44 (*m*/*z*) were identified as major contributors from the evolved gases and volatiles, and determined to be H_2_, CH_4_, CO, and CO_2_, respectively. The produced Ni@G samples were labeled as Ni@G-600 °C, Ni@G-700 °C, Ni@G-800 °C, and Ni@G-900 °C.

### 2.5. Temperature-Programmed Catalytic Decomposition of Methane (TPCDM)

Temperature-programmed catalytic decomposition of methane (TPCDM) was performed in a fixed-bed reactor system equipped with on-line mass spectroscopy. The Ni@G sample (10 g) was first purged by flowing with Ar (50 mL/min) at room temperature for 0.5 h. Then, the reactant gas mixture of 100 mL/min methane and 50 mL/min argon were introduced. The reactor was heated from 25 to 900 °C at a heating rate of 10 °C/min, during which the *m*/*z* intensities at 2 (H_2_), 16 (CH_4_), 28 (CO), and 44 (CO_2_) in the effluents were recorded by the online mass spectrometer.

### 2.6. Catalytic Testing for Methane Decomposition

The catalytic performance for methane decomposition of each catalyst was tested in a ceramic 1-inch (O.D.) tubular fixed-bed reactor at a temperature range of 700–900 °C. The preferred amount of catalyst (i.e., 2.5, 5, 10, 15, and 20 g) was loaded in the middle of the reactor. Catalytic testing was performed using 100 mL/min CH_4_ and 50 mL/min Ar. Outlet gases were analyzed using the online mass spectrometer. The argon in the reaction gas was used as a diluent and as an internal analysis standard. Pressure of the reactor system was recorded by a pressure transducer and compared with time on stream. The differential pressure sensor was connected to the reactor to measure the difference in gas pressure before and after the reactor and this pressure difference (Δ*P*) was monitored and recorded.

Catalytic activity was evaluated in terms of methane conversion. We defined methane conversion (M_conv_) as

M_conv_ (%) = (M_in_ − M_out_)/M_in_) × 100% (4)

where M_in_ represents the total quantity (moles) of methane fed into the reactor and M_out_ represents the quantity (moles) of methane out of the reactor.

The solid carbon in the fresh Ni@G and the Ni@G sample after CDM process was measured by TGA. Twenty milligrams of the sample (20 mg) was put on a ceramic sample pan for TPO analysis in a Shimadzu TGA-50H instrument. By heating the sample under high flow of air (100 mL/min) from room temperature to 800 °C using a 10 °C/min heating ramp rate, the weight change was recorded as related to carbon burning. C_1_ is defined as the carbon in the Ni@G sample originated from lignin, and C_m_ is the carbon in the sample contributed by methane after CDM reaction.

### 2.7. Characterization

Nickel content (wt%) of the graphitized Ni-lignin samples was measured using an inductively coupled plasma-optical emission spectrometer (ICP-OES, Agilent 5800, Santa Clara, CA, USA). Ni@G samples were mineralized with nitric acid before ICP-OES measurement. Surface area measurement of the Ni@G samples was determined using an automatic adsorption unit (Autosorb–1, Quantachrome, Boynton Beach, FL, USA). The specific surface areas were calculated using the Brunauer–Emmett–Teller (BET) analysis method to the respective N_2_ adsorption isotherms. X-ray powder diffraction (XRD) patterns of the samples were obtained using a Rigaku Ultima III X-ray Diffraction System (Rigaku, TheWoodlands, TX, USA). The morphology of the samples was investigated with a JEOL 6500F Field Emission Scanning Electron Microscope (SEM, Peabody, MA, USA). The sample particle sizes were examined with a JEOL JEM-100CX II Transmission Electron Microscope (TEM, Peabody, MA, USA). Raman Spectroscopy measurements were carried out using a Jobin-Yvon microspectrometer (Edison, NJ, USA) equipped with an excitation laser source emitting at 514 nm and an incident power around 1 mW on a thin surface. Twenty spectra were collected for each sample.

## 3. Results and Discussion

### 3.1. Catalytic Carbonization of Ni-Lignin Composite to Few-Layer Graphene Encapsulated Nickel Nanoparticles (Ni@G)

**Figure 1:**
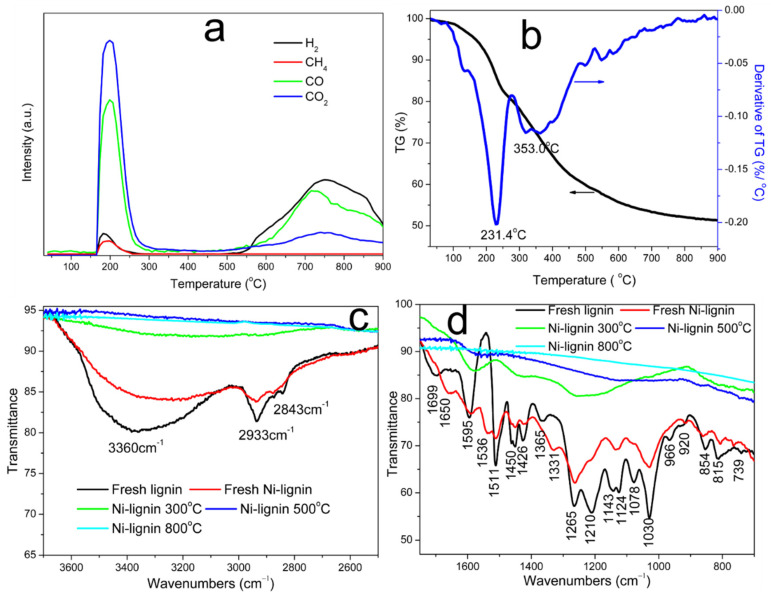
Gas evolution profiles of temperature-programmed carbonization (**a**), TG/DTG curves (**b**) of the Ni-lignin sample under argon atmosphere, and FTIR spectra (**c**,**d**) of the kraft lignin and the Ni-lignin composite thermally treated at different temperatures: 300 °C, 500 °C, and 800 °C, respectively.

Figure 1a shows the trends of vent gas species (CO_2_, CO, CH_4_, and H_2_) during temperature-programmed catalytic carbonization of the Ni-lignin sample. Significant amounts of CO_2_, CO, CH_4_, and H_2_ were released from the Ni-lignin sample between the temperatures of 165 and 300 °C. This implied that kraft lignin was catalytically decomposed by nickel ions. Carboxyl, carbonyl, and ether groups in the phenylpropane side chains will decompose and release as CO_2_, CO, and CH_4_ when heating Ni-lignin at the lower temperature. CO_2_ released in the low temperature range is attributed to the catalytic decomposition of carboxyl groups in kraft lignin [17] and/or catalytic oxidation of functional groups in lignin by Ni^2+^ ions [16]. The CO formation in the low temperature range is mainly contributed by decomposition of carbonyl and/or catalytic oxidation of functional groups in lignin by Ni^2+^ ions, lignin char is formed after the decomposition of the most of functional groups in lignin. In the second stage, the formed char was catalytically carbonized to carbon from 300 °C to 1000 °C. The strong CO peak at 555 °C was assigned to ether decomposition, implying that nickel ions promoted the hydrolysis of the ether groups. The weak intensity CO_2_ peak at 490 °C was assigned to the carbothermal reduction of nickel oxide. Hydrogen evolution was observed when the temperature was above 490 °C and was assigned to the cracking of –CH_x_ (x = 1–3) groups promoted by nickel metal. In the present work, nickel oxide dissolved in the lignin matrix was first reduced by surface functional groups of the lignin, then the reduced metallic nickel reacted with amorphous carbon to form Ni@C nanostructures (NiO + active functional groups → Ni + CO_2_ + CO + H_2_O).

TG (thermogravimetric analysis) and DTG (derivative of TG) curves of the Ni-kraft lignin sample are plotted in Figure 1b. As shown, the catalytic decomposition process could be divided into five stages corresponding to five mass loss steps shown in the TG curves: (1) water evaporation, (2) decomposition of nickel nitrate and de-polymerization of side chain structures, (3) decomposition of aromatic ring structures and formation of lignin char, (4) reduction of nickel oxide by active carbon species in lignin char, and (5) catalytic graphitization of lignin char by nickel particles [18].

The FTIR spectra of kraft lignin is plotted in Figure 1c,d and compared to that of the Ni-lignin composites. A strong peak at 3368 cm^−1^ and two small peaks at 2937 and 2841 cm^−1^ are assigned to stretching vibrations of hydroxyl, methyl and methylene groups in lignin, respectively [19,20]. The IR peak at 1710 cm^−1^ is associate with carbonyl groups. Peaks at 1597, 1511, and 1417 cm^−1^ are due to stretching vibrations of aromatic rings in lignin [13]. The stretch absorptions of C-C, C-O, and C=O are located at 1265 and 1215 cm^−1^, respectively [16,19]. The IR band at 1078 cm^−1^ is associated with C-O deformation of secondary alcohol of the side chains [21].

FTIR spectra of the fresh Ni-lignin composites is plotted in Figure 1c,d and compared to that of kraft lignin. Due to the formation of bonds between nickel ions and the functional groups in lignin molecules, the intensity and location of IR bands were found to be reduced and/or shifted in the Ni-lignin composite. FTIR spectra of Ni-lignin samples heated at 300 °C, 500 °C, and 800 °C are also presented in Figure 1c,d. Most of the functional groups in kraft lignin were decomposed after heating the sample at 300 °C as most of the FTIR bands disappeared or significantly decreased, with the exception of aromatic ring structures, which were partially broken when the sample was thermally treated at 300 °C. Almost all the FTIR bands disappeared after thermally treating the samples at 500 °C and 800 °C, indicating the structure of kraft lignin was decayed due to catalytic carbonization. FTIR results in Figure 1c,d are in good agreement with TPD-MS (Figure 1a) and TGA (Figure 1b) results.

### 3.2. Characterization of Ni@G Samples

Inductively coupled plasma–optical emission spectrometry (ICP-OES) was used to measure the nickel content in the Ni@G samples and the results are listed in Table 1. The Ni contents of 10% Ni-lignin composites, were 22.8%, 23.6%, 24.9%, and 25.5% for Ni@G samples carbonized at 600 °C, 700 °C, 800 °C, and 900 °C, respectively. The nickel contents of 2.5%, 5%, 10%, 15%, and 20% Ni-lignin composites were 6.9%, 11.8%, 25.5%, 34.2%, and 43.5%, respectively, in Ni@G samples carbonized at 900 °C. The average crystallite size of metallic Ni in the Ni@G products was calculated using the Scherrer equation.

Table 1 indicates that BET surface areas were 57.4, 83.7, 117.5, 91.6, and 73.5 m^2^/g for kraft lignin materials with nickel loadings of 2.5%, 5%, 10%, 15%, and 20%, respectively. This indicates that with increased nickel loading, surface areas increased from 2.5% to 10% loading, and decreased with 15% to 20% loading.

**Figure 2:**
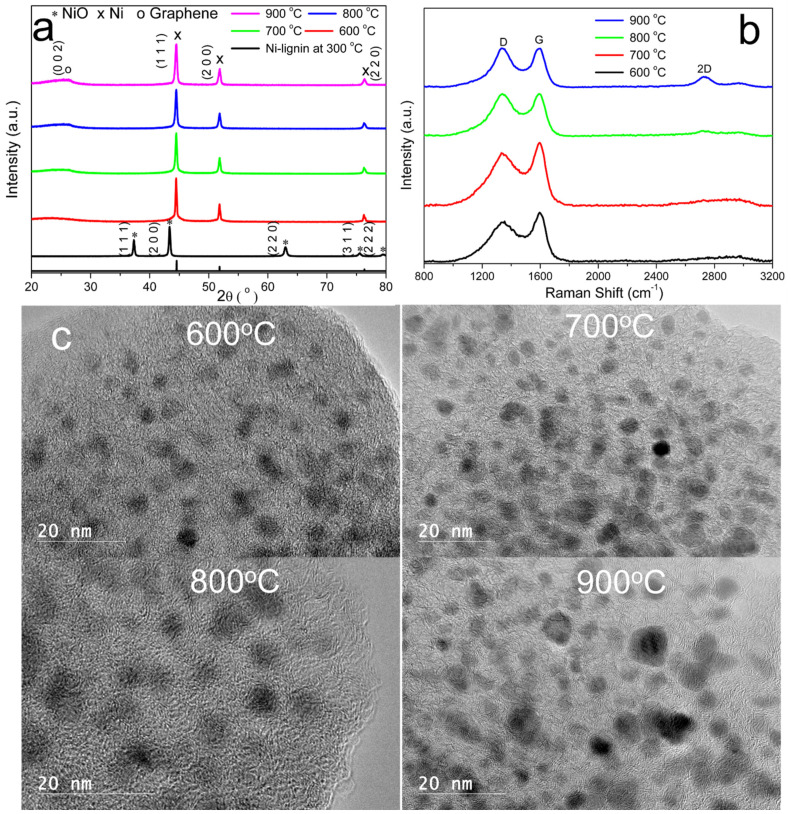
X-ray diffraction (XRD) patterns (**a**), Raman spectra (**b**), and HRTEM images (**c**) of 10% Ni-lignin composite catalytically graphitized under argon atmosphere at different temperatures.

Figure 2a shows XRD patterns of the calcined Ni-lignin sample and the carbonized 10% Ni-lignin samples in the 2θ range of 20–80°. The XRD pattern of the calcined Ni-lignin shows diffraction peaks at 37.3°, 43.3°, 63.1°, 75.5°, and 79.6°, which are assigned the (111), (200), (220), (311), and (222) planes of NiO. The peaks observed at 2θ of 44.5°, 51.8°, and 76.4° are characteristic of fcc nickel phase, corresponding to (111), (200), and (220) planes, indicating the Ni has a polycrystalline structure; the XRD profile clearly shows the nickel phase is majority face-centered cubic structure. At 2θ = 26° a single peak attributed to (002) graphite is present for the samples carbonized at 700 °C, 800 °C, and 900 °C. X-ray photoelectron spectroscopy (XPS) provides information about the chemical state of elements. XPS spectra of the calcined Ni-lignin and the 10% Ni-lignin carbonized at 800 °C are plotted in Figure S1. Ni2p3/2 peaks for both samples were very weak, possibly because the nickel particles were encapsulated in carbon or graphene.

Raman spectroscopy can identify the presence of graphite and disordered amorphous carbon in the samples. Figure 2b shows Raman spectra of the carbonized 10% Ni-lignin samples under different temperatures. The *A_D_*/*A_G_* values of the prepared samples were 1.50, 1.46, 1.39, and 1.29 for Ni-lignin samples carbonized at 600 °C, 700 °C, 800 °C, and 900 °C, respectively; therefore, the degree of graphitization of these four samples was in the order of 600 °C < 700 °C < 800 °C < 900 °C.

#### High-Resolution Transmission Electron Microscopy

The microstructure and morphology of 10% Ni-lignin samples graphitized at temperatures between 600 °C and 900 °C were investigated by HRTEM. Figure 2c shows dark colored Ni nanoparticles (XRD results in Figure 2a) embedded in the light-colored amorphous lignin char matrix in the Ni-lignin-600 sample. One to two layers of graphitic carbon were observed to form around nickel nanoparticles of the Ni-lignin sample carbonized at 700 °C, indicating the minimum temperature for the formation of Ni@G structure was 700 °C; these Ni@G structures were surrounded by amorphous carbon. Similar Ni@G structures formed for the Ni-lignin sample carbonized at 800 °C, where the graphitic carbon around nickel nanoparticles increased to 2–4 layers and the amorphous carbon converted to the turbostratic stacking structure [22]. When the carbonization temperature increased to 900 °C, the graphitic shell increased to 3–8 layers and almost all the amorphous carbon was converted to the turbostratic stacking structure.

**Figure 3:**
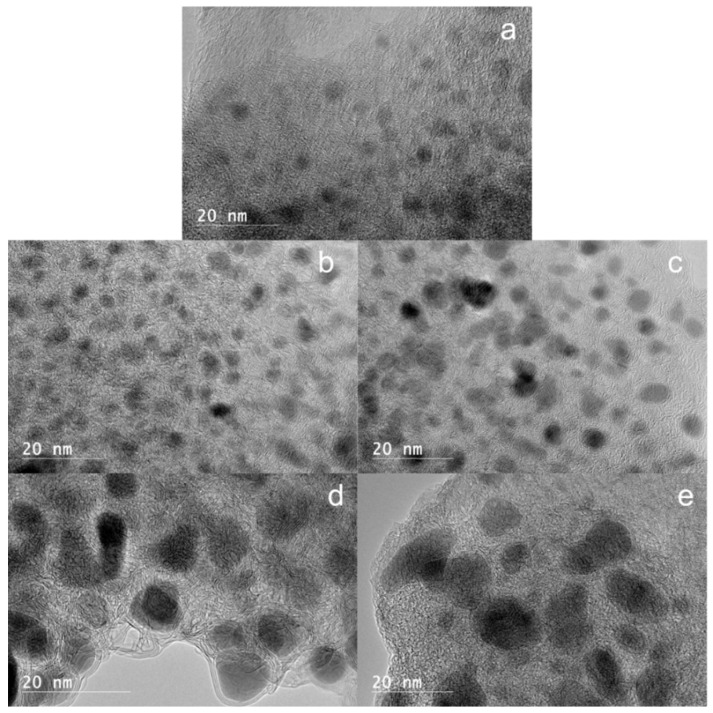
HRTEM images of Ni-lignin composites with different nickel loadings ((**a**) 2.5%; (**b**) 5%; (**c**) 10%; (**d**) 15%; (**e**) 20%) catalytically graphitized under argon atmosphere at 900 °C.

The effect of nickel loading on the morphology of the Ni@G samples was investigated using high resolution transmission electron spectroscopy (HRTEM). The images of Ni@G structures from 2.5%, 5%, 10%, 15%, and 20 wt% Ni-lignin carbonized at 900 °C are shown in Figure 3. The nickel particles in Ni@G samples from 2.5%, 5%, and 10 wt% Ni-lignin composites were homogenous and well dispersed in the turbostratic stacking structure; these Ni@G nanoparticles were 2–8 nm in diameter. The morphologies of Ni@G samples from 15% and 20% Ni-lignin composites were significantly different from those observed in 2.5%, 5%, and 10% Ni-lignin composites in terms of particle sizes and sample phases.

HRTEM micrographs (Figure 3d,e) show that increasing the nickel loading percentage to 15 and 20 wt% resulted in metal agglomeration and lower dispersion of the Ni@G nanoparticles in the carbon matrix. This results in a negative effect on the activity and stability of the catalyst sample.

### 3.3. Catalytic Decomposition of Methane over Ni@G Samples

#### 3.3.1. Temperature-Programmed Catalytic Decomposition of Methane (TPCDM) over Ni@G Nanoparticles

**Figure 4:**
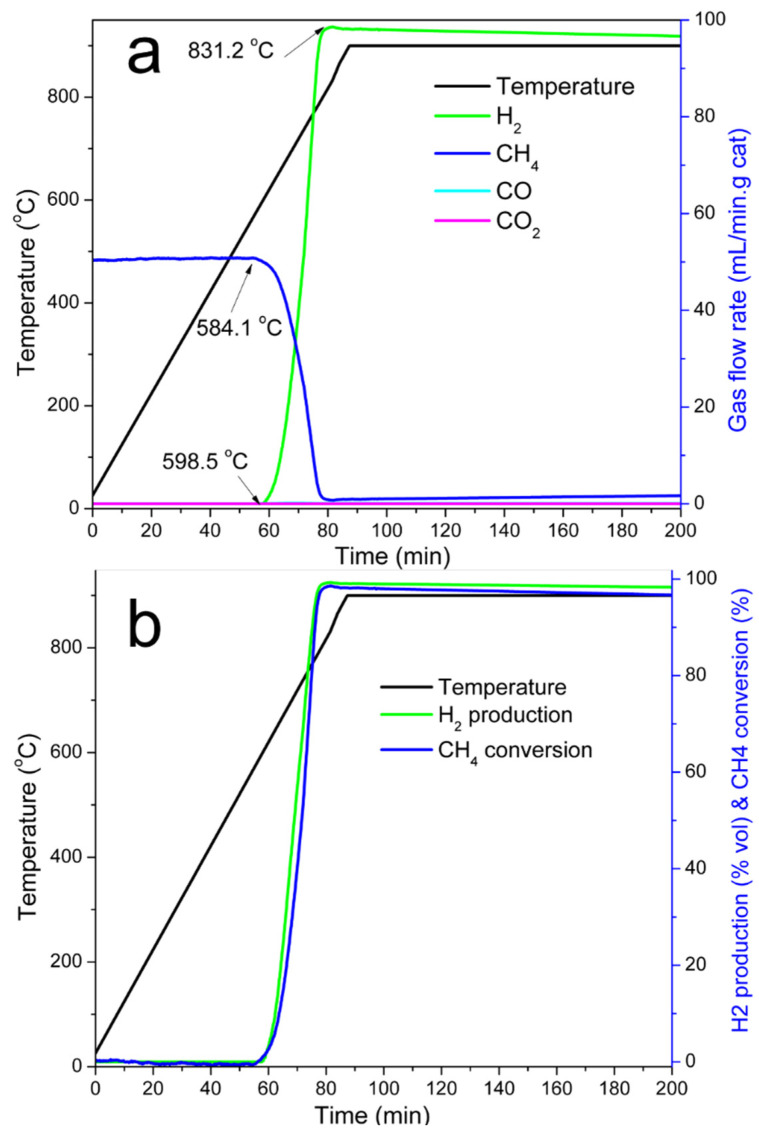
TPCDM over 25.5% Ni@G. (**a**). Gas flow rates vs. time, (**b**). H_2_ vol% and CH_4_ conversion rate (%) vs. time. Experimental conditions: 10 g catalyst, heating rate 10 °C/min, temperature range 25–900 °C, and flow rate of CH_4_ 100 mL/min.

Temperature programmed reaction experiments were carried out to validate if the Ni@G materials were active for methane decomposition. Temperature-programmed CDM reaction was performed by flowing Ar-CH_4_ mixture (50 mL/min Ar-100 mL/min CH_4_) through a fixed-bed reactor loaded with 10 g of 25.5% Ni@G material. The reactor was ramped with a heating rate of 10 °C/min to 900 °C. The flow rates of methane and hydrogen in the vent gas, the conversion rate of methane, and the production rate of hydrogen were plotted in Figure 4. CH_4_ decomposition over 25.5% Ni@G was initiated at 584.1 °C (Figure 4). Ni@G exhibited very poor activity at low temperature and only a small amount of methane was consumed at 584.1 °C; hydrogen production was initially detected at 598.5 °C. The methane consumption rate accelerated with increase of heating temperature and reached the maximum at 831.2 °C.

The structures and C/Ni mass ratios of graphene products from Ni@G samples are observed to depend on the structure of Ni@G samples. Ni@G prepared at different carbonization temperatures, using different nickel contents, or different process parameters like CDM reaction temperature, mass of the loaded Ni@G samples, or reaction time, will influence the structure. The effects of the Ni@G structures and CDM reaction process parameters on graphene products were investigated in the CDM process. The structures of Ni@G, CDM reaction process conditions, and the structure of the graphene products are listed in Table 2.

##### Effect of Carbonization Temperature

**Figure 5:**
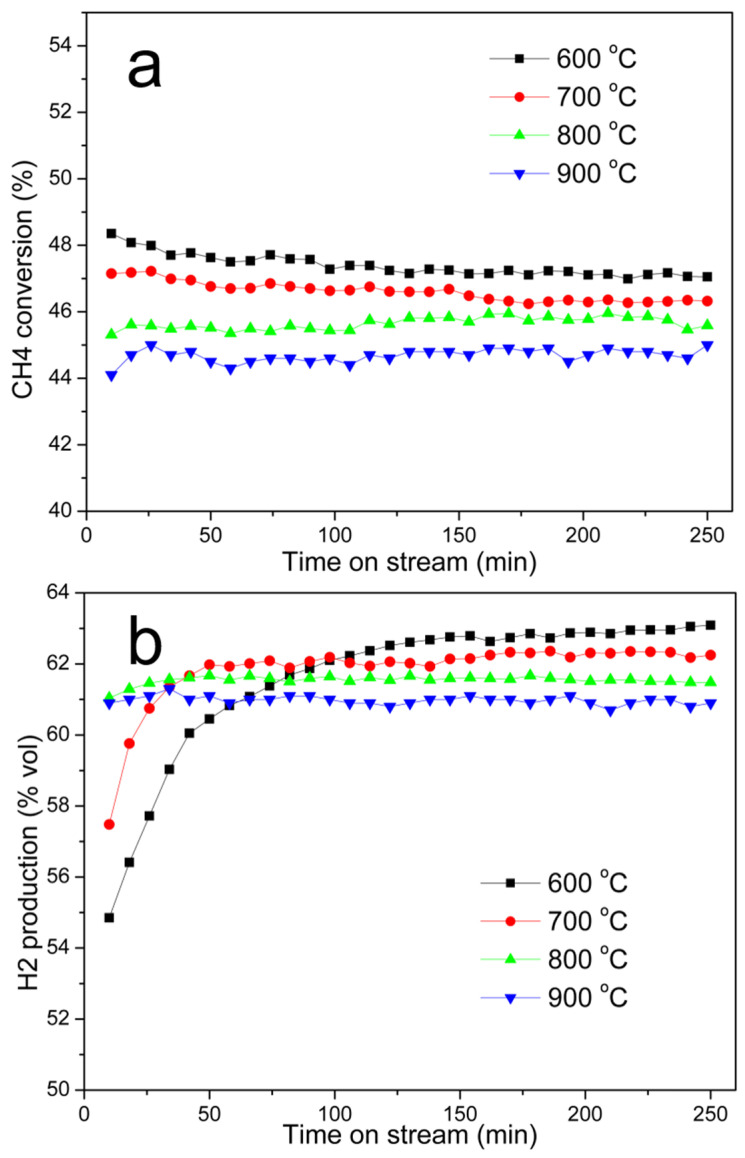
CH_4_ conversion (**a**) and H_2_ production (**b**) with Ni@G catalysts carbonized at different temperature: 10 g catalyst was loaded in the reactor, testing temperature: 800 °C; gas flow rate: 100 mL/min.

Catalytic activity of Ni@G materials from the Ni-lignin composite carbonized under different temperatures was investigated with the CDM reaction, and the results are shown in Figure 5. Figure 5a exhibits the activity of Ni@G prepared at the carbonization temperature of 600 °C, 700 °C, 800 °C, and 900 °C. The results show the conversion of CH_4_ was in the following order: Ni@G-600 °C > Ni@G-700 °C > Ni@G-800 °C > Ni@G-900 °C. The Ni@G-600 °C and Ni@G-700 °C catalysts show higher initial methane conversion, and the conversion rate decreases slowly with time on stream. The Ni@G-900 °C sample demonstrates the lowest activity; however, the methane conversion rate remains steady under the testing conditions.

There are several possible explanations for why the Ni@G-600 °C and Ni@G-700 °C exhibit high initial methane conversion rates. (1) Due to lower carbonization temperatures, nickel nanoparticles in both samples are relatively small: 5.7 nm for Ni@G-600 °C and 8.5 nm for Ni@G-700 °C (Table 1). Furthermore, HRTEM images (Figure 2) show these nickel nanoparticles as uniformly distributed in the amorphous carbon matrix and no graphene (carbon) shells are observed to encapsulate the nickel nanoparticles; therefore, they are active and ready to decompose methane molecules. (2) Because of lower carbonization temperatures, lignin char in the Ni@G-600 °C and Ni@G-700 °C samples are only partially carbonized, and the carbon-based structures in both samples are mainly amorphous carbon. Amorphous carbon structures have proved to be the most active carbon-based materials for catalytic decomposition of methane [4,23]. (3) Ni@G-600 °C and Ni@G-700 °C samples were prepared at the carbonization temperatures of 600 °C and 700 °C, respectively. Lignin char was not fully carbonized in either sample, therefore, significant amounts of oxygen-containing functional groups remained. At the beginning of the CDM reaction, amorphous carbon structures in Ni@G-600 °C and Ni@G-700 °C samples will continue the carbonization process and produce Cox (CO and CO_2_) (Figure S2). Initial hydrogen production rates (Figure 5b) of the Ni@G-600 °C and Ni@G-700 °C samples are lower due to the production of Cox (CO and CO_2_).

##### Effect of the Reaction Temperature

**Figure 6:**
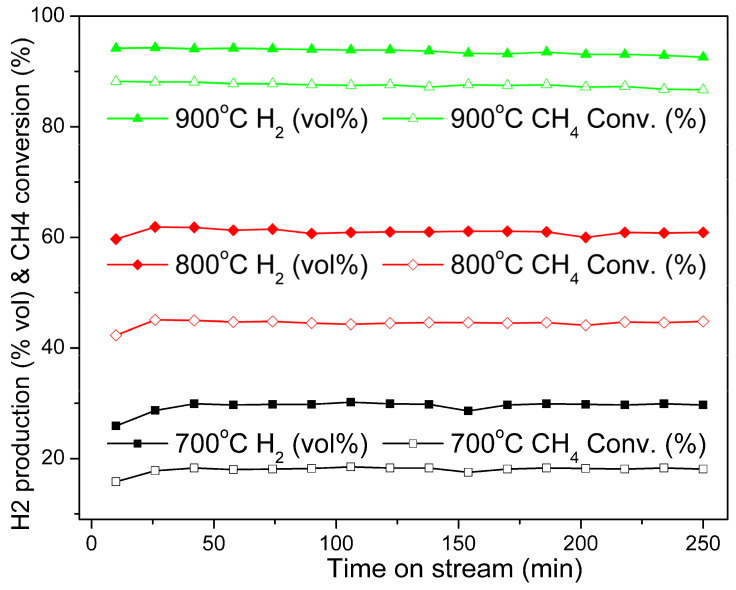
Effect of the reaction temperature on catalytic decomposition of methane over 25.5% Ni@G nanoparticles. Catalyst used: 10 g; gas flow rate: 100 mL/min; testing temperature: 700 °C, 800 °C, and 900 °C.

The activity of methane decomposition over 25.5% Ni@G at different reaction temperatures (700 °C, 800 °C, and 900 °C) as a function of time on stream was studied, and the results are displayed in Figure 6. Only hydrogen and unreacted methane were detected in the vent gases. As shown in Figure 6, CH_4_ conversion and H_2_ production rates increase with increase of the reaction temperature from 700 to 900 °C. An initial methane conversion of 15.8% was observed for the reaction at 700 °C, and the methane conversion rate reached a steady state of 18.3% after 45 min. The initial methane conversion rate of 42.3% was observed at 800 °C, and it increased to a stable level of 45% after 25 min. The methane conversion rate was 88.1% from the beginning at 900 °C and there was no induction time present at the higher reaction temperature.

##### Effect of the Amount of Catalyst

**Figure 7:**
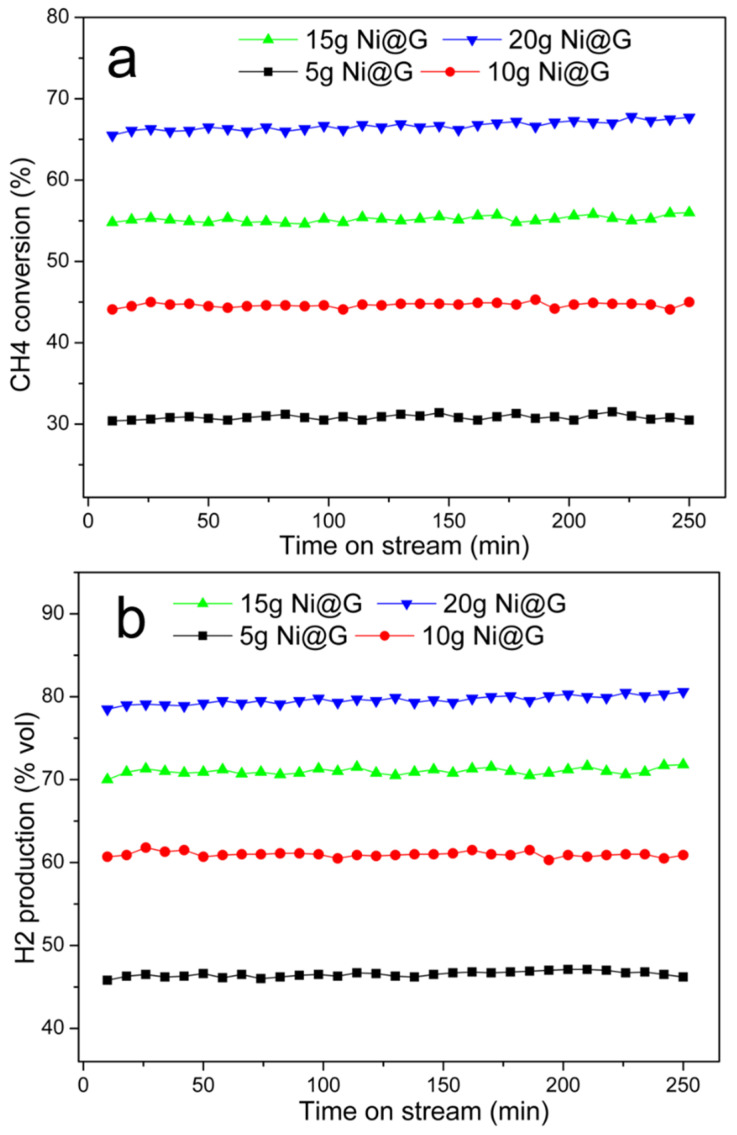
CH_4_ conversion and H_2_ production with different amounts (5g, 10 g, 15 g, and 20 g) of 25.5% Ni@G catalyst loaded in the reactor. (**a**). CH_4_ conversion rate (%) vs. time, (**b**). H_2_ vol% in vent gas vs. time. Testing temperature: 800 °C; gas flow rate: 100 mL/min.

The effects of Ni@G sample mass were examined for the CDM reaction. Figure 7 illustrates the methane conversion and hydrogen production rates when the reaction is performed with 5, 10, 15, and 20 g of 25.5% Ni@G at 800 °C. The results revealed that CH_4_ conversion and hydrogen production rate increased with increase of the catalyst mass due to longer residence time of the methane when more catalyst is loaded in the reactor. It was also noticed that methane conversion and hydrogen production rates remained constant, and no deactivation of the catalyst was observed under the operating conditions.

##### Effect of Ni@G Nickel Content

**Figure 8:**
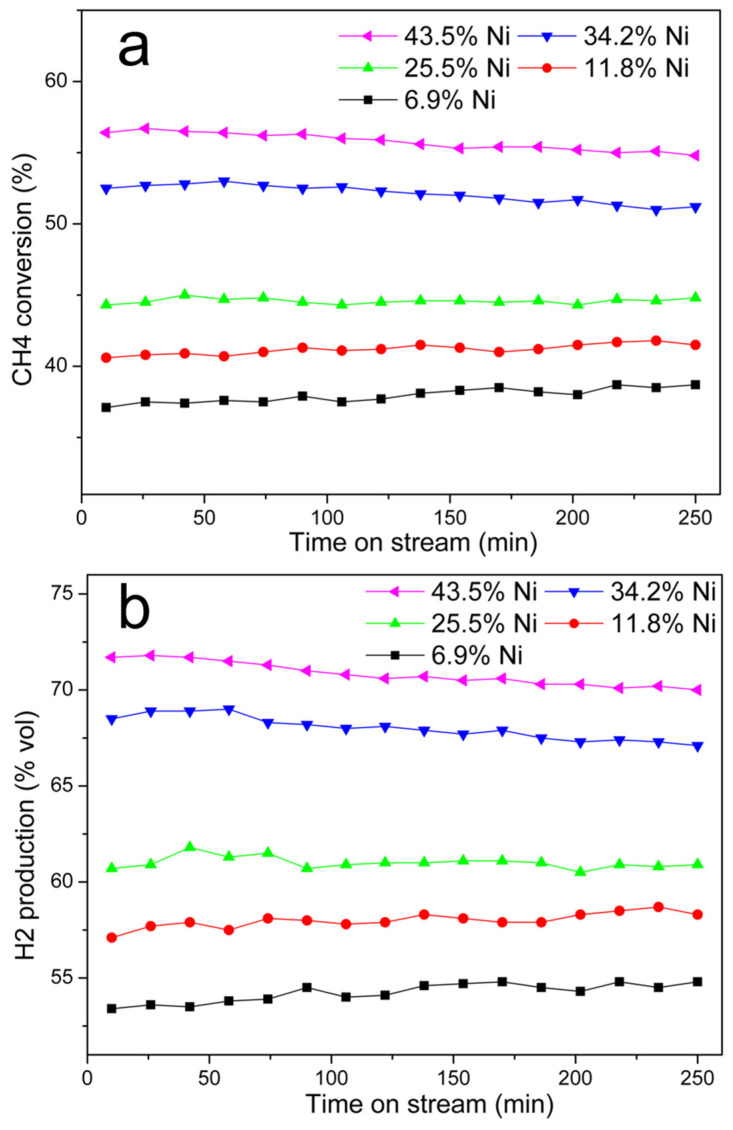
CH_4_ conversion and H_2_ production over Ni@G samples with different nickel contents (6.9 wt%, 11.8 wt%, 25.5 wt%, 34.2 wt%, and 45.3 wt%). (**a**). CH_4_ conversion rate (%) vs. time, (**b**). H_2_ vol% in vent gas vs. time. Testing temperature: 800 °C; catalyst used: 10 g; gas flow rate: 100 mL/min.

Five Ni@G samples with different nickel contents were obtained by graphitization of Ni-lignin composites with nickel loading of 2.5%, 5%, 10%, 15%, and 20%. After catalytic graphitization at 900 °C for 1 h, the nickel contents in the corresponding Ni@G samples were 6.9%, 11.8%, 25.5%, 34.2%, and 43.5%, respectively. The activity of Ni@G for catalytic decomposition of methane at 800 °C for 250 min is plotted in Figure 8. As shown in Figure 8, both the CH_4_ conversion rate and the H_2_ production rate increase with the increase of nickel contents in Ni@G samples. Ni@G samples with 6.9%, 11.8%, and 25.5% Ni contents showed relatively lower initial methane conversion and hydrogen production rates, but good stability under the reaction conditions. Ni@G samples with 34.2% and 43.5% Ni contents exhibited high initial activity but the methane conversion and hydrogen production rates decreased gradually with time-on-stream. The decrease in activity may be caused by the agglomeration of the Ni particles at high carbonization temperature, resulting in large Ni particle size and a lowering of the surface area of the nickel catalyst. Furthermore, the low carbon content in high Ni loading samples may also contribute to the deactivation of the catalyst during CDM reaction since graphene is another active part of the Ni@G sample in the decomposition of methane.

##### Stability of the Catalyst

**Figure 9:**
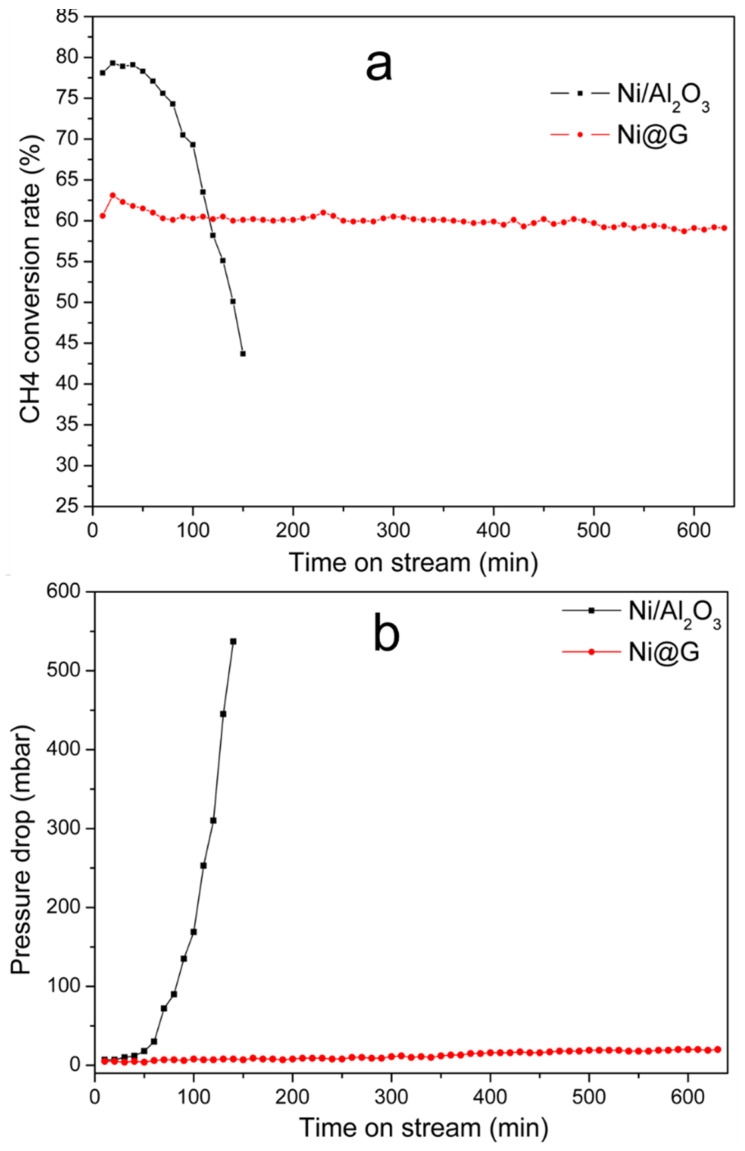
Comparison of 25.5% Ni@G and 10% Ni/Al_2_O_3_ for CDM reaction: (**a**) CH_4_ conversion rate (%) and (**b**) pressure drop across the catalyst bed. Reaction conditions: 800 °C; catalyst used: 10 g; gas flow rate: 100 mL/min.

To evaluate the stability of the Ni@G materials, 25.5% Ni@G and 10% Ni/Al_2_O_3_ were compared in the CDM reaction. Both the CH_4_ conversion rate and the pressure drop (Δ*P*) across the catalyst beds are presented as a function of time-on-stream (TOS) in Figure 9. The 10% Ni/Al_2_O_3_ catalyst exhibited unstable activity. The initial methane conversion rate was 78.1% after 10 min, then increased to 79.5% after 20 min, then kept at ~79.0% after 40 min TOS. After 50 min TOS, the catalyst began to show continuous deactivation and after 150 min, the conversion rate decayed to 43.7%. The pressure drops across the Ni/Al_2_O_3_ catalyst bed also changed with TOS. The initial Δ*P* of Ni/Al_2_O_3_ was 7 mbar at 10 min TOS. It increased to 18 mbar after 50 min TOS. The pressure drops then increased very quickly, reaching 537 mbar at 150 min TOS; the reactor was clogged and the CDM reaction was paused. The higher initial methane conversion of Ni/Al_2_O_3_ catalyst could be attributed to the larger number of Ni sites [2,24]. However, because of high CDM activity, significant carbon deposits were formed over the catalyst and resulted in the reactor plugging.

The Ni@G catalyst showed relatively lower activity compared to the Ni/Al_2_O_3_ catalyst. The CH_4_ conversion rate was 60.5% at 10 min TOS and increased to 63.1% after 20 min TOS. Gradually, the methane conversion rate became stable at about 60% during 600 min TOS. The pressure drop for the Ni@G catalyst was very low. Initially, the Δ*P* of Ni@G was 5 mbar at 10 min TOS and increased slowly to 20 mbar after 600 min TOS. The catalytic stability may be attributed to the nickel dispersion in the Ni@G sample. The cracked graphene shells may also participate in the CDM reaction as the vacancy defects and edges in the cracked graphene shells can serve as the active sites for methane decomposition [25,26,27]. The vacancies and edge carbon atoms with free bonds in the cracked graphene shells tend to react with methane molecules by catching the carbon atoms to stabilize their structures. The active sites in vacancy defects will be consumed over TOS, while the carbon atoms on the edges will continuously be regenerated with new atoms decomposed from methane molecules through CDM reaction. Therefore, CDM activity over the Ni@G sample remained stable during the 600 min of TOS.

### 3.4. Sample Characterization after CDM Reaction

**Figure 10:**
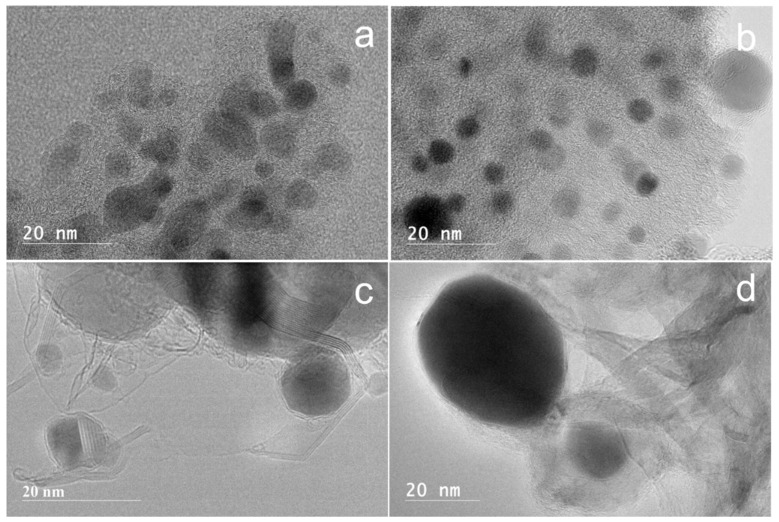
HRTEM images of the Ni@G samples after CDM reaction for 1 h at different reaction temperature: (**a**) 600, (**b**) 700, (**c**) 800, and (**d**) 900 °C.

HRTEM images of the Ni@G samples after CDM reaction for 1 h at different reaction temperatures (600, 700, 800, and 900 °C) are showed in Figure 10. There is no obvious change in the Ni@G sample cracked at 600 °C; the Ni@G particles keep their original structures, i.e., the nickel particles are captured in 1–5 layers of graphene shells which are not yet cracked (Figure 10a). As the temperature increased to 700 °C, the HRTEM image shows the graphene shells of the Ni@G on the sample surface are cracked and partially peeled off the nickel cores due to the CDM reaction (Figure 10b). When the temperature increases to 800 °C, the core-shell structures of Ni@G are cracked and the graphene shells are skinned off the nickel cores to form graphene nanoplatelets (Figure 10c). The size of the nickel particles was significantly increased to 10–50 nm due to merging of the naked nickel cores. HRTEM image of the Ni@G samples after CDM reaction for 1 h at 900 °C (Figure 10d) shows more large graphene nanoplatelets are formed and the nickel particles are sintering to even larger particles.

**Figure 11:**
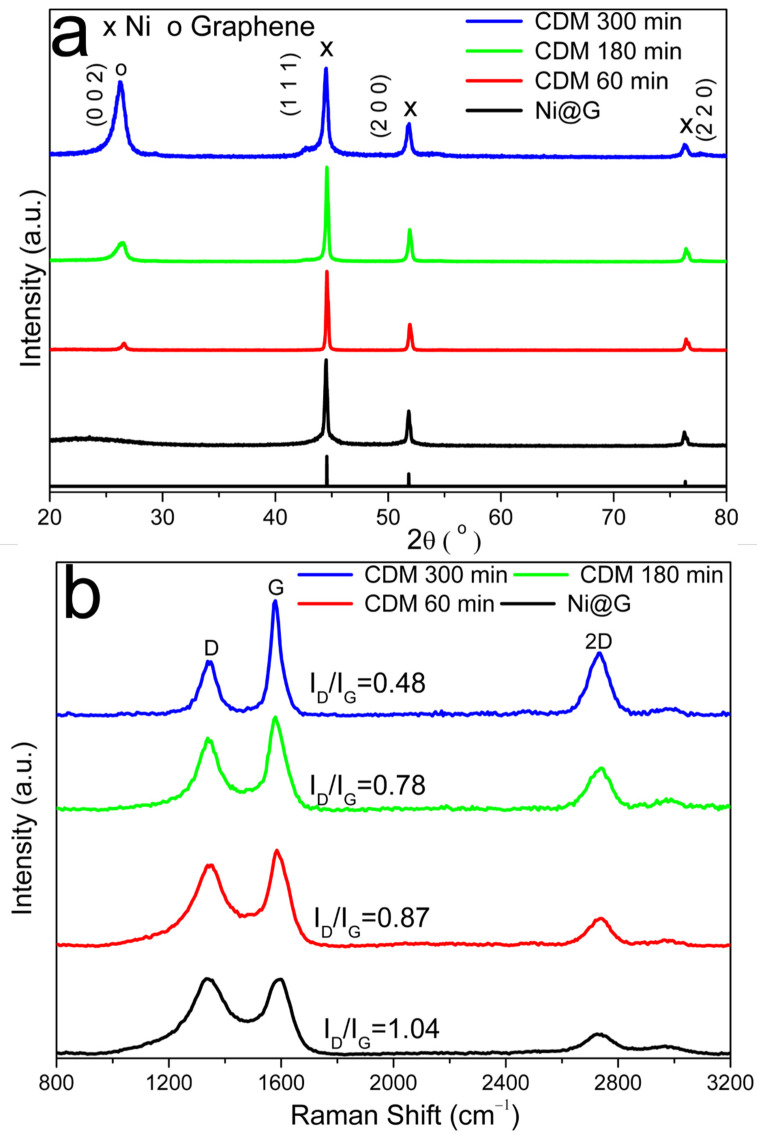
XRD patterns (**a**) and Raman spectra (**b**) after CDM reaction at 800 °C with different reaction time.

Figure 11a shows the XRD patterns of the products of Ni@G after CDM reaction for different reaction times (60 min, 120 min, 180 min, and 300 min) at 800 °C. The relative intensity of the graphene diffraction peak (2θ = 26.5°) increases with increased reaction time. A weak and flat diffraction peak at ~26.5° is observed for the graphene sample at 60 min, and the peak becomes strong and sharp with increase of reaction time. This indicates the graphene nanoplatelets grow with improved quality with prolonged reaction time.

Quality of the graphene produced from Ni@G through CDM reaction at 800 °C with different reaction times was analyzed by Raman spectroscopy (Figure 11b). Quality of grown graphene materials was assessed by the intensity ratio between bands D and G (I_D_/I_G_). The I_D_/I_G_ ratio decreased with the prolonged reaction time, indicating a decrease in defects present in the graphene materials. It is also noticed that the I_2D_/I_G_ ratio decreased with increase of reaction time, indicating there are more graphene layers in graphene samples produced with longer reaction time.

**Figure 12:**
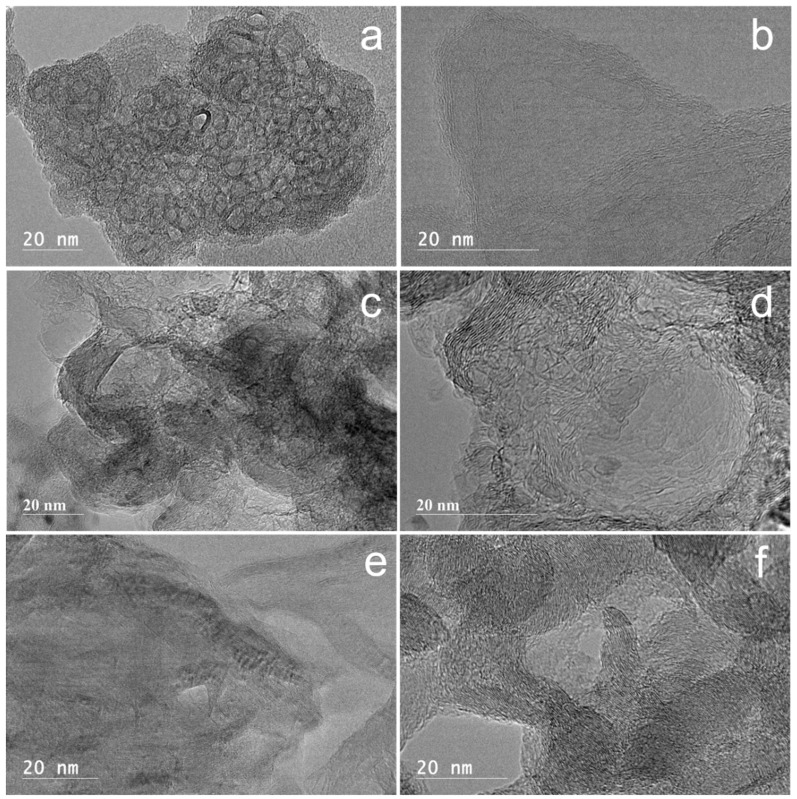
HRTEM images of the purified carbon materials from Ni@G samples after CDM reaction at 800 °C with different reaction time: Ni@G without CDM reaction (**a**), 30 min (**b**), 60 min (**c**), 120 min (**d**), 180 min (**e**), and 300 min (**f**).

Fresh Ni@G and Ni@G samples after CDM reaction with different reaction times were boiled in 5 M HNO_3_ solution to remove nickel particles and other inorganic impurities. This was followed by rinsing the sample with deionized water and oven drying at 105 °C overnight. HRTEM image (Figure 12a) of the purified fresh Ni@G sample shows it is mainly composed of empty graphene shells after nickel particles were removed. Almost all the nickel particles are removed from the Ni@G samples from different reaction times after acid purification. After CDM reaction for 30 min, the graphene shells in the Ni@G samples are cracked and peeled off the nickel particles and are randomly distributed in various directions. Methane molecules decompose to carbon atoms and hydrogen over both the surface of the nickel particles and the cracked graphene shells. The carbon atoms deposited over nickel particles will form graphene nanoplatelets while the carbon atoms deposited over the cracked graphene shells may fill vacancy defects in the graphene shells or react along the edges of the graphene shells to joint or merge these graphene shell units together. However, few or no joint reactions occur among them due to the short time process, therefore, fluffy graphene is the main structure in the products. Fluffy graphene is usually made up of 1 to 5 layers of graphene with an in-plane size of 30–50 nm (Figure 12b). HRTEM image (Figure 12c) of the purified graphene sample after 60 min of CDM reaction shows the formation of 3D fluffy graphene structures. This suggests welding or jointing reactions happened between the cracked graphene shells during the CDM process; the welding action mainly involves bonding along the edges of the cracked graphene shell units. HRTEM image (Figure 12d) reveals graphene nanoplates are the main products of the Ni@G after 120 min CDM reaction. The cracked graphene shells are combined along both the edges and the in-plane directions of units to form graphene nanoplates which show a thickness between 1 and 2 nm and an in-plane size of 100–300 nm. When the CDM reaction runs over Ni@G samples for a prolonged time, i.e., 180 min and 300 min (Figure 12e,f, respectively), 3D graphitic nanochips are the main structure of the graphene products. These graphitic structures are produced when more cracked graphene shells are welded along the horizontal edge and the in-plane directions due to prolonged reaction time. The graphitic nanochips show an in-plane size of 0.5–2 microns with a thickness range of 5–10 nm.

#### Possible Decomposition Mechanism of Methane over Ni@G

Nickel is known to be an excellent active metal component for the decomposition of methane [18]; however, Ni@G exhibited very poor activity in the decomposition of methane at low temperature. This is because most of the nickel particles are encapsuled by several layers of graphene, and it is very difficult for CH_4_ to diffuse and penetrate through the graphene shell if there are no defective cracks [26]. Fortunately, there are some naked nickel particles or Ni@G with defects in the graphene shell. As the reaction temperature is increased, a small amount of methane will diffuse along the cracks in the graphene shell of Ni@G and adsorb onto the surface of the nickel core, followed by the dissociation reaction of methane to hydrogen and carbon [26,28]. The carbon atoms will deposit on the nickel surface while hydrogen atoms may either combine and desorb as H_2_ to enter the gas phase or diffuse and dissolve into the nickel core of the Ni@G [26,28]. As the smallest molecule and an excellent decarburization reagent, the hydrogen molecules can easily diffuse and penetrate through the few-layer graphene shell then react with carbon dissolved in the nickel particles to form methane. With increasing of the heating temperature, more methane decomposition reactions occur over Ni@G. Consequently, more hydrogen penetrates the graphene shells of Ni@G and decarburizes the dissolved carbon in nickel cores to methane. Due to its larger size compared to hydrogen, methane molecules from the decarburization process cannot diffuse through the few-layer graphene shells and are trapped in the interface between the graphene shell and the nickel core. This results in the build-up of methane pressure inside the Ni@G structure which causes the outer graphene shell to break up. With the graphene shell cracked, the nickel core will be naked and exposed to the gaseous phase and more nickel active sites will be available for decomposition of methane. Simultaneously, the cracked graphene shells will serve as a carbon-based catalyst for decomposition of methane. The carbon atoms from methane decomposition will serve as an atomic glue to join the cracked graphene shells and can also deposit to vacancy or defect sites in the cracked graphene shells to improve the quality of the graphene product. The graphene shell cracking process can explain the required induction period at lower reaction temperatures (Figure 6) as it will take some time for methane molecules to diffuse and dissociate over nickel core particles. Raman results (Figure 11b) indicate a decrease in defects and more layers of graphene produced in the graphene material samples produced with longer reaction time. HRTEM images (Figure 12) show the cracked graphene shells are combined along both the edges and the in-plane directions to form graphene nanoplates which become larger and thicker after prolonged CDM reaction time.

Overall, two reaction processes may simultaneously occur during the CDM reaction over Ni@G: (1) the cracking of Ni@G by methane molecules at the beginning of the reaction. and (2) after the cracking step, the exposed nickel particles and the cracked graphene shells will serve as the active catalyst components for CDM reaction.

## 4. Conclusions

Ni-lignin nanocomposites were prepared from nickel nitrate and kraft lignin. These Ni-lignin nanocomposites were catalytically graphitized to few-layer graphene-encapsulated nickel nanoparticles (Ni@G). Ni@G nanoparticles were used for catalytic decomposition of methane (CDM) to produce COx-free hydrogen and graphene nanoplatelets. Ni@G catalysts exhibit high activity and long-term stability for use in the CDM reaction.

During the CDM reaction process, the graphene shells of the Ni@G nanoparticles were cracked and peeled off the nickel cores at high temperature. Both the exposed nickel nanoparticles and the cracked graphene shells may participate in the CDM reaction, making Ni@G samples highly active for CDM reaction. The vacancy defects and edges in the cracked graphene shells serve as active sites for methane decomposition and the edges are continuously regenerated through the CDM reaction. Therefore, Ni@G samples remained stable for CDM reaction during 600 min TOS. Graphene nanoplatelets, fluffy graphene, 3D fluffy graphene, and 3D graphitic nanochips were formed as the main solid products in the CDM reaction over Ni@G catalyst.

## Figures and Tables

**Table 1:** Catalytic graphitization of Ni-lignin composites to graphene-encapsulated nickel nanoparticles (Ni@G). The “@G” indicates the graphene (carbon)-encapsulation.

Ni-Lignin Precursor	Carbonization Temperature (°C)	Ni Content in Ni@G (%) ^a^	Ni Particle Size (nm) ^b^	Surface Area (m^2^/g) ^c^
10% Ni-lignin	600	22.8	5.7	79.1
10% Ni-lignin	700	23.6	8.5	92.6
10% Ni-lignin	800	24.9	9.4	108.2
10% Ni-lignin	900	25.5	10.2	117.5
2.5% Ni-lignin	900	6.9	4.9	57.4
5% Ni-lignin	900	11.8	7.3	83.7
15% Ni-lignin	900	34.2	27.6	91.6
20% Ni-lignin	900	43.5	32.5	73.5

a. Measured by ICP-OES, b. Calculated by XRD results, c. Measured by BET.

**Table 2:** Graphene-based materials from CDM reaction over graphene-encapsulated nickel nanoparticles (Ni@G). The “@G” indicates the graphene (carbon)-encapsulation.

Nickel Content in Ni@G (%)	Ni@G Used (g)	Reaction Temperature (°C)	Reaction Time (min)	Ni Particle Size in the Product (nm)	Surface Area (m^2^/g)	C_l_/Ni Ratio (g/g)	C_m_/Ni Ratio (g/g)	C_t_/Ni Ratio (g/g)
22.8	10	800	250	7.9	69.3	-	-	5.82
23.6	10	800	250	10.7	75.1	-	-	5.63
24.9	10	800	250	17.5	81.9	-	-	5.35
25.5	10	800	250	27.2	87.2	2.92	4.73	5.29
25.5	10	700	250	15.8	101.3	2.92	0.95	3.87
25.5	10	900	250	29.6	75.3	2.92	4.46	7.39
25.5	5	900	250	-	-	2.92	3.26	6.18
25.5	10	800	250	-	-	2.92	2.36	5.29
25.5	15	800	250	-	-	2.92	1.93	4.85
25.5	20	800	250	-	-	2.92	1.73	4.65
6.9	10	800	250	7.5	53.7	13.49	6.79	20.29
11.8	10	800	250	12.7	62.5	7.47	4.54	12.01
34.2	10	800	250	35.8	73.8	1.92	1.96	3.88
43.5	10	800	250	47.1	58.2	1.30	1.66	2.96
25.5	10	800	30	13.6	-	2.92	0.30	3.22
25.5	10	800	60	16.7	-	2.92	0.59	3.51
25.5	10	800	120	19.3	-	2.92	1.15	4.08
25.5	10	800	180	25.2	-	2.92	1.70	4.62
25.5	10	800	300	30.5	-	2.92	2.80	5.73

C_l_: carbon contributed from lignin; C_m_: carbon from decomposition of methane; C_t_ = C_l_ + C_m_.

## Data Availability

Not applicable.

## Supplementary Materials

The following supporting information can be downloaded. Figure S1: The XPS spectra for Ni2p3/2 states of the calcined Ni-lignin at 300 °C and the carbonized Ni-lignin at 800 °C. Figure S2: TPCDM over Ni@G of Ni-lignin carbonized at 600 °C.
