# Supplementary material for: Production of COx-Free Hydrogen and Few-Layer Graphene Nanoplatelets by Catalytic Decomposition of Methane over Ni-Lignin-Derived Nanoparticles
Source: Molecules. 2022 Jan 14;27(2):503. doi: 10.3390/molecules27020503 (PMC8777900; doi:10.3390/molecules27020503)
Supplement: Supplementary file 1 [file molecules-27-00503-s001.zip › molecules-1501864-supplementary.pdf]

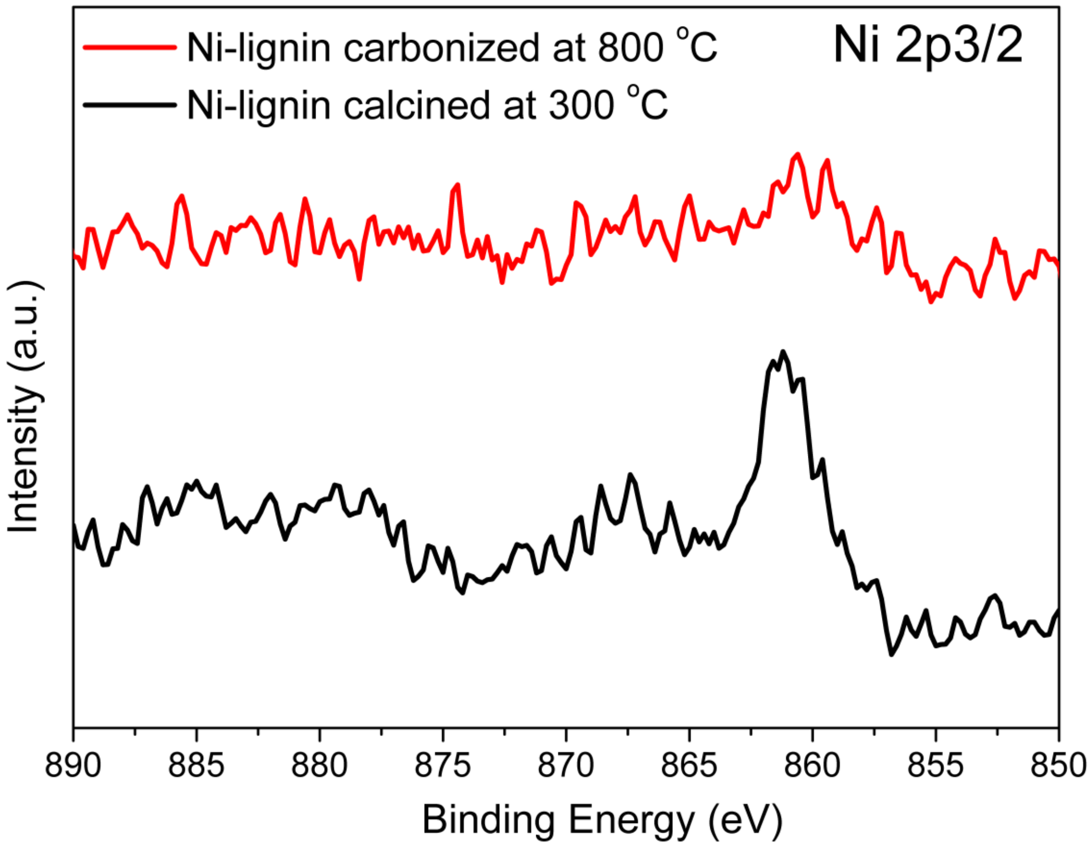

Figure S1. The XPS spectra for Ni2p3/2 states of the calcined Ni-lignin at 300°C and the carbonized Ni-lignin at 800°C. Measurement of photoelectron spectra was conducted by using a PHI model 1600 XPS instrument, with Al Kα X-ray source, and the spectra was calibrated with reference to the C 1s level at 284.8 eV.

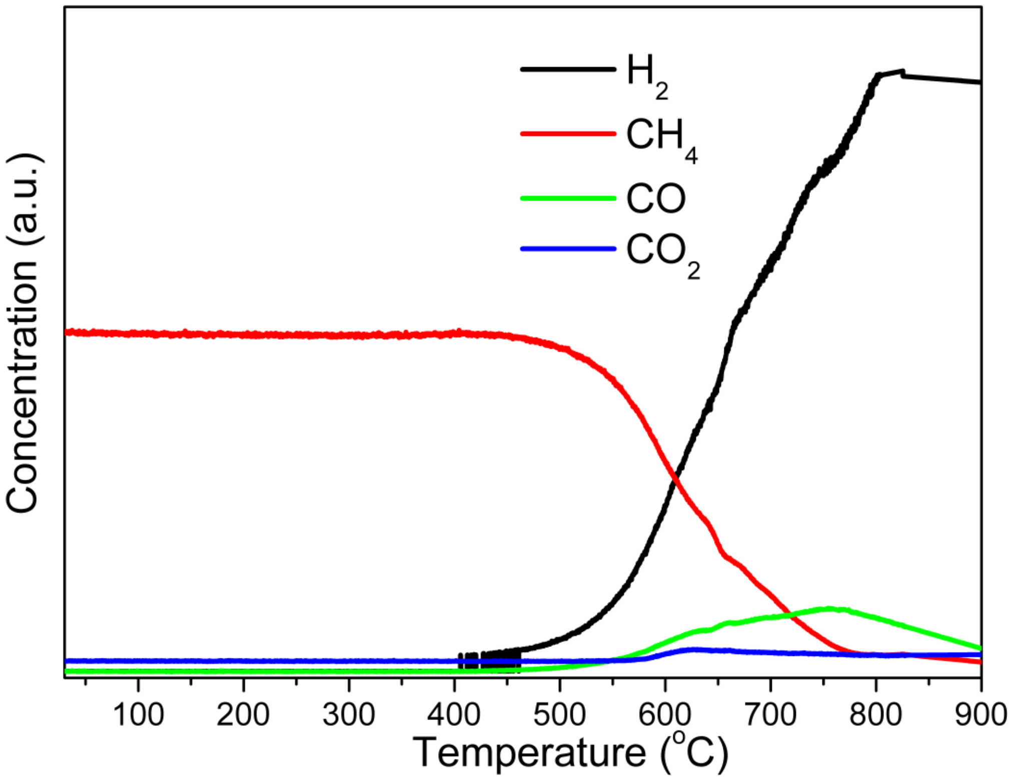

Figure S2. TPCDM over Ni@G of Ni-lignin carbonized at 600°C. Experimental conditions: 10 g catalyst, heating rate 10 °C/min, temperature range 25-900°C, and flow rate of CH_4 100 mL/min.
